# Microsatellite-high intrahepatic cholangiocarcinoma with favorable treatment outcome using pembrolizumab

**DOI:** 10.1007/s12328-025-02103-4

**Published:** 2025-03-04

**Authors:** Shigeru Horiguchi, Hironari Kato, Kazuya Miyamoto, Kosaku Morimoto, Akihiro Matsumi, Hiroyuki Terasawa, Yuki Fujii, Kazuyuki Matsumoto, Takehiro Tanaka, Motoyuki Otsuka

**Affiliations:** 1 Department of Gastroenterology and Hepatology, Okayama University Hospital, 2-5-1 Shikata-cho, Kita-ku, Okayama-City, Okayama 700-8558 Japan; 2 Department of Pathology, Okayama University Hospital, Okayama, Japan

**Keywords:** Microsatellite instability (MSI)-high, Tumor mutation burden (TMB)-high, Intrahepatic cholangiocarcinoma, Comprehensive genome profiling

## Abstract

Intrahepatic cholangiocarcinoma has a poor prognosis. In unresectable cases, the survival period is short despite combination therapy with cytotoxic anticancer agents and immune checkpoint inhibitors. The usefulness of immune checkpoint inhibitors against malignant tumors with microsatellite instability-high (MSI-H) mutations was shown in the KEYNOTE158 study; however, data for intrahepatic cholangiocarcinoma are insufficient. In the present case, a 65-year-old man with intrahepatic cholangiocarcinoma and lymph node metastasis could not be treated with a combination of gemcitabine, CDDP, and S-1. A comprehensive cancer genomic profiling (CGP) test showed *MLH1* pathogenic mutation and MSI-H. When pembrolizumab was administered, the tumor shrinkage effect was rapidly observed, which was sustained even after 30 months. No pathogenic mutations were observed in the germline test, and MSI-high was considered to be due to the *MLH1* pathogenic mutation occurring sporadically in somatic cells. MSI-H intrahepatic cholangiocarcinoma is extremely rare. However, because pembrolizumab is expected to be effective, CGP testing should be actively performed.

## Introduction

Intrahepatic cholangiocarcinoma is a primary malignant tumor of the liver with an extremely poor prognosis and a high frequency in East Asia. Gemcitabine (GEM) and cisplatin (CDDP) combination therapy is the standard treatment for patients with unresectable tumors and postoperative recurrence [1]. In Japan, GEM, CDDP, and S-1 combination therapy have been reported to have better results than GEM and CDDP combination therapy [2], but the results are not satisfactory. In addition, GEM, CDDP, and PD-L1 antibody therapy has been newly approved for biliary tract cancer [3] and is expected to expand in the future. However, the report is not sufficient to expect a breakthrough in terms of overall survival and progression-free survival.

Pembrolizumab has been approved for use in microsatellite instability-high (MSI-H) solid tumors. This treatment is based on the KEYNOTE158 study [4] and is promising for cross-organ precision medicine. However, MSI-H in biliary tract cancer is rare, being approximately 1.7–3.1% [5–7], and there are few reports on treatment [8–13].

Herein, we report a case of MSI-H intrahepatic cholangiocarcinoma refractory to GEM, CDDP, and S-1 combination therapy in a patient who received pembrolizumab, achieved a partial response on imaging, and continued treatment for 30 months.

## Case report

**Fig. 1:**
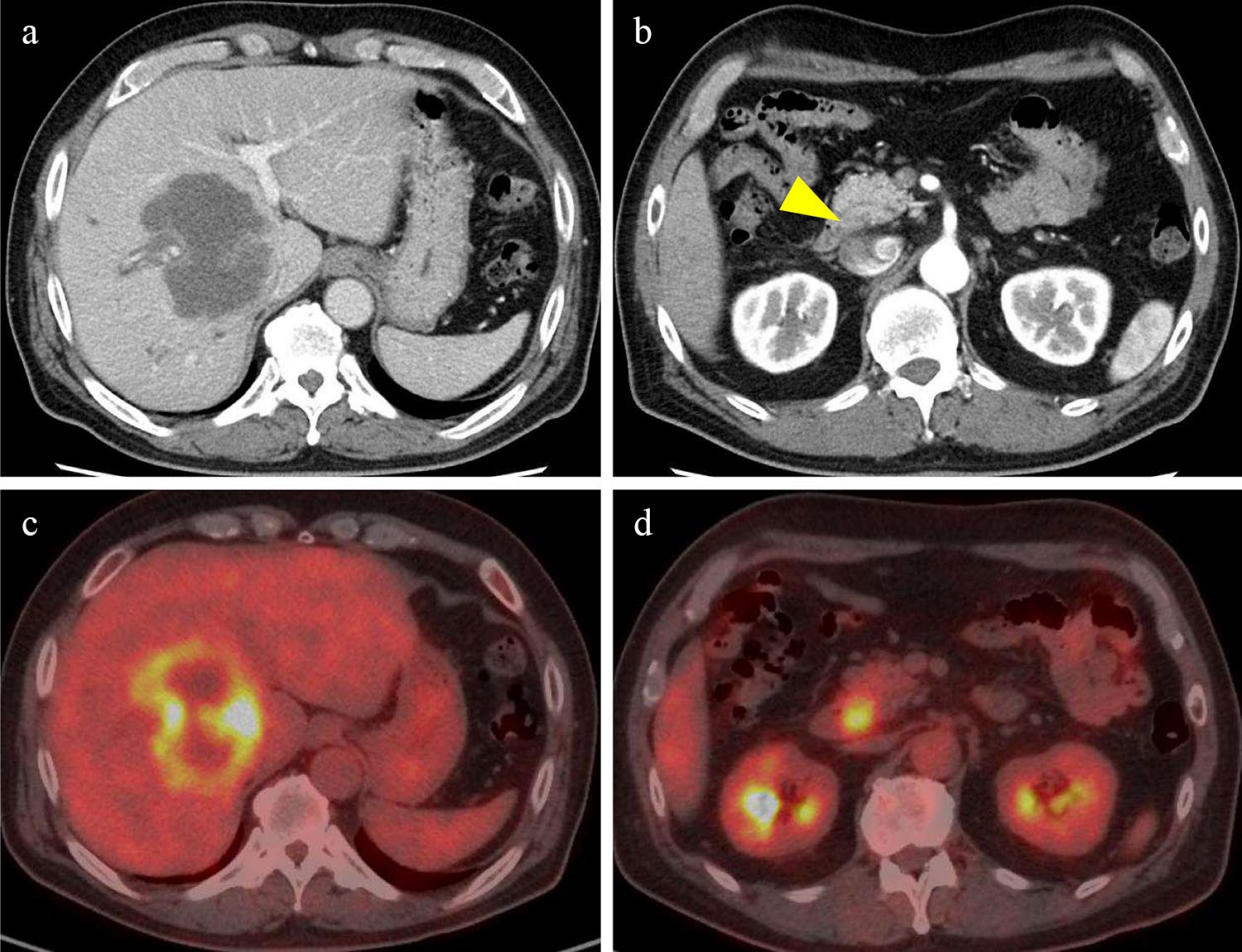
Contrast-enhanced computed tomography (CT) (**a**, **b**) and positron emission tomography-CT (**c**, **d**) images. **a** Intrahepatic cholangiocarcinoma with minimal enhancement in the right lobe of the liver. **b** Swollen lymph nodes are observed behind the pancreatic head. **c** Fluorodeoxyglucose (FDG) collection is observed in the tumor of the right lobe of the liver. **d** FDG accumulation is observed in the lymph nodes of the pancreatic head, which is considered a metastasis

A 65-year-old man was referred to our hospital because of an intrahepatic tumor identified during a medical examination. The patient’s Eastern Cooperative Oncology Group performance status score was 0. He had an uneventful family history and a past medical history of hypertension. Laboratory examinations revealed high concentrations of γ-glutamyl transpeptidase (520 U/L), alkaline phosphatase (362 U/L), carcinoembryonic antigen (CEA, 983 ng/mL), and cancer antigen (CA19-9, 85,964 U/mL). Tests for hepatitis B surface (HBs) antigen, HBs antibody, and hepatitis C virus antibody were all negative. Contrast-enhanced computed tomography (CECT) showed a solitary 72-mm-diameter tumor deep in the anterior segment of the liver, with low enhancement and delayed appearance of contrast (Fig. 1). In addition, swelling of the pancreatic head lymph nodes was observed, and fluorodeoxyglucose (FDG)-positron emission tomography (PET) showed accumulation of FDG at the same sites, suggesting lymph node metastasis (Fig. 1). We performed an ultrasound-guided liver biopsy to obtain tissue samples. We used an anterior segmental approach to access the tumor in the right lobe of the liver. Using a Top aspiration biopsy needle 21G × 150 mm (New Majima Needle, TOP corporation, Tokyo, Japan) (outer needle diameter 0.80 mm), we performed 5 needle passes and confirmed tissue collection. Pathological diagnosis of the specimens revealed adenocarcinoma. Esophagogastroduodenoscopy or colonoscopy showed no abnormal findings, and based on CT and PET-CT examination results, we diagnosed intrahepatic cholangiocarcinoma.

**Fig. 2:**
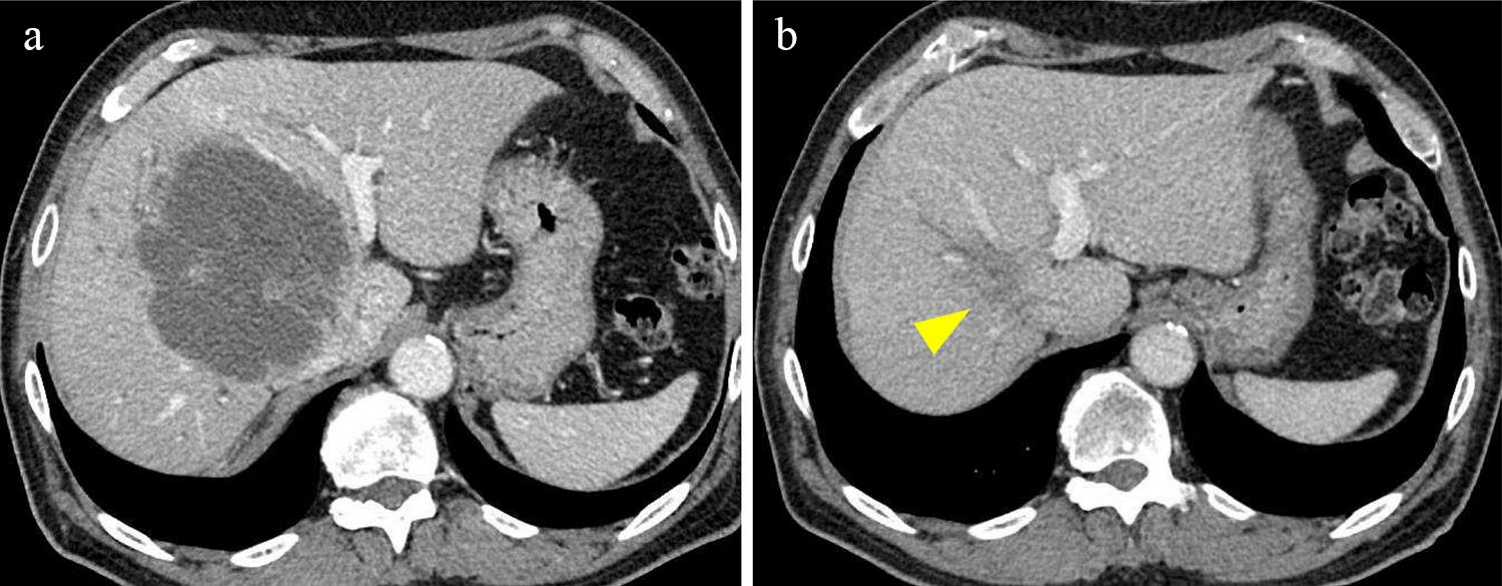
Contrast-enhanced computed tomography image. **a** Image of progressive disease after GCS therapy. Portal vein umbilicus slightly shifted to the left due to an enlarged tumor lesion. **b** After three 40 cycles of pembrolizumab, the primary tumor (yellow arrow) showed a remarkable reduction. GCS, gemcitabine (GEM) + concurrent cisplatin (CDDP) + adjuvant tegafur-gimeracil-oteracil (S-1)

As this case involved intrahepatic cholangiocarcinoma with lymph node metastasis, curative resection was impossible. We decided to perform anticancer drug treatment with GEM + CDDP + S-1 (GCS). However, 3 months after the start of GCS therapy, CECT revealed an increase in intrahepatic lesions, indicating progressive disease (Fig. 2a).

A comprehensive cancer genomic profiling test using FoundationOne CDx was performed. The results are summarized in Table 1. The genomic purity assessment was 22.2%. MSI-H, tumor mutation burden-high (TMB-H) (> 10 muts/Mb), and many other gene mutations were observed. Based on the results of genomic profiling, pembrolizumab was administered at a dose of 200 mg/body once every 3 weeks. Nine months after the start of administration, type 1 diabetes mellitus developed as an immune-related adverse event, and insulin therapy was initiated; however, no other notable adverse events were observed. CECT performed every 3 months showed rapid shrinkage of the intrahepatic lesions (Fig. 2b), accompanied by normalization of tumor markers.

**Table 1:** Comprehensive genomic profiling test results

Microsatellite status MSI-High			Tumor mutational burden 29 muts/Mb		
Gene	Variant site	VAF	Gene	Variant site	VAF
*MLH1*	K196fs*	0.49	*PIK3CA*	D350G	0.076
*KRAS*	G12D	0.21	*PIK3CA*	R88Q	0.13
*CIC*	S1117fs*34	0.054	*PTCH1*	S1203fs*52	0.068
*CTCF*	E363fs*5	0.25	*QKI*	K134fs*14	0.21
*EP300*	M1470fs*26	0.032	*RNF43*	G659fs*41	0.43
*FBXW7*	R465C	0.21	*TP53*	Splice site 920-1G > A	0.24
*MLL2*	L656fs*274	0.26	*TP53*	R273H	0.25
*MLL2*	P496fs*3	0.22	*JAK3*	Q39fs*108	0.24
*MLL2*	R755fs*175	0.21	*PBRM1*	I279fs*4	0.41

*VAF* variant allele frequency

This table does not include variant of unknown significance

**Fig. 3:**
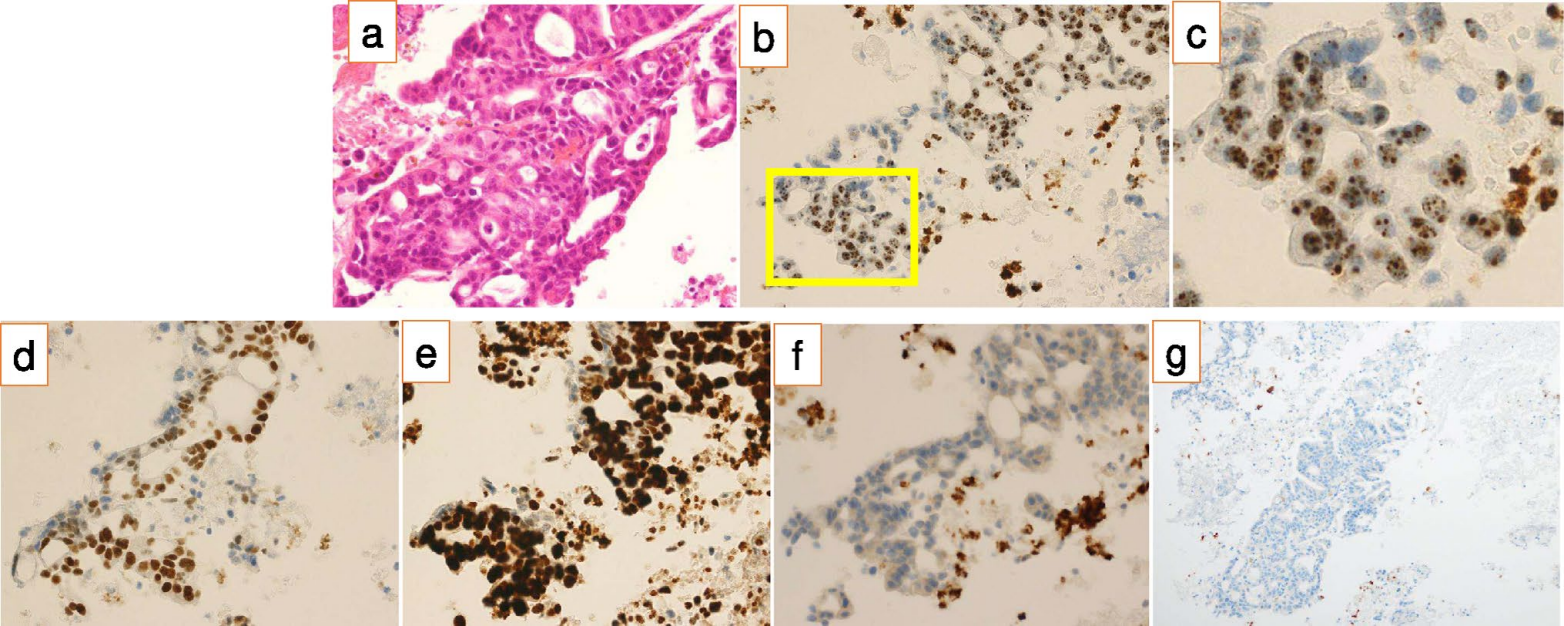
Histochemical analyses with immunostainings. **a** Hematoxylin and eosin staining showing adenocarcinoma with fibroplasia and infiltrative growth of the atypical cuboidal epithelium in the form of irregular-to-fused glands (*X400*). **b** Immunostaining image of MSH1 in the lesion. Figure 3c is a magnified image of the yellow box (*X400*). c. Magnified image of the yellow box in Fig. 3b. It was judged to be a false-positive image due to dot-like staining. **d** MSH2 immunostaining image was determined to be positive (*X400*). e. MSH6 immunostaining image was determined to be positive (*X400*). **f** PMS2 immunostaining image was determined to be negative (*X400*). **g** PD-L1 immunostaining image was determined to be negative (*X100*)

Since the MLH1 gene mutation (K196fs*) is classified as pathogenic in ClinVar and MSH-H was detected in the comprehensive cancer genome profiling test, the possibility of Lynch syndrome was considered, and immunostaining for mismatch repair-related proteins was performed. Immunostaining for MLH1, MSH2, MSH6, and PMS2 revealed the absence of MLH1 and PMS2 expression (Fig. 3). At first glance, MLH1 expression was positive; however, fine dot-like staining was observed when magnified. Based on studies describing the characteristics of MLH1 false-positive findings [14,15], it was judged as a characteristic finding of false positive and an MLH1 protein deficiency. After further genetic counseling, germline mismatch repair genetic testing (ACTRisk™ Care, ACTmed corporation, Tokyo, Japan) was performed to establish a definitive diagnosis of Lynch syndrome. This testing examined all exonic regions of the MLH1 gene, not just the mutation site detected in CGP testing. No mutations were found in any of the germline genes, and the MSI-H in this case was diagnosed as being caused by somatic mutations.

Thirty months after treatment initiation, CT shows the tumor maintains significant reduction with no evidence of regrowth. CA19-9 levels remain within normal range. The patient’s performance status is 0, and treatment continues.

## Discussion

The efficacy of pembrolizumab for MSI-H solid tumors was reported in KEYNOTE158 [4]. The response rate in all carcinomas was 34.4%, including 9.9% complete responses; in 22 cases of biliary tract cancer, the response rate reached 40.9%, demonstrating efficacy. In our case of intrahepatic cholangiocarcinoma, MSI-H was also detected, and pembrolizumab was administered, showing a marked long-term effect.

The KEYNOTE-158 study demonstrated that TMB-H could be a useful predictive biomarker for response to pembrolizumab in solid tumors [16]. Among the 790 evaluable patients, those with TMB-H status (≥ 10 mutations per megabase) showed a significantly higher objective response rate of 29% (95% CI: 21–39) compared to 6% (95% CI: 5–8) in non-TMB-H patients. The durability of response was also notable, with the median duration of response not reached in the TMB-H group versus 33.1 months in the non-TMB-H group. Importantly, this predictive value of TMB-H was observed independent of PD-L1 expression and was not driven by outcomes from any particular tumor type. These results suggest that TMB-H status could be a useful pan-tumor biomarker to identify patients who may benefit from pembrolizumab monotherapy across various solid tumor types.

In this case, although there was no past or family history of colorectal cancer, the MSI was high, and the variant allele frequency of the MLH1 mutation was 0.49; therefore, considering the possibility of Lynch syndrome was necessary. Hence, both MLH1 and PMS2 were negative when immunostaining for mismatch repair-related gene proteins was performed. A germline test was performed to make a definitive diagnosis of Lynch syndrome based on immunostaining results. Germline examination did not reveal any abnormalities in any of the mismatch repair-related genes, suggesting sporadic MSI-H intrahepatic cholangiocarcinoma. TMB-H was also detected simultaneously.

MSI-H is rare in biliary tract cancer, reported in approximately 1.7–3.1% of cases [5–7]. The clinical features of MSI-H biliary tract cancer have not been reported in large numbers, and the clinicopathological or radiological features remain unclear [17]. To date, case reports of intrahepatic bile ducts diagnosed as MSI-H and treated with PD-1 antibody (Table 2) showed no consistent trends with respect to age, sex, or disease stage [8–13]. As for tumor markers, CA19-9 was substantially high in Toshida’s report; however, in Kai’s and Ikeda’s reports, there were cases of mild elevation, which was unclear in some cases, making detailed examination difficult (Table 2). The therapeutic effect was successful in all cases and the progression-free survival was prolonged.

**Table 2:** Patients treated with pembrolizumab for MSI-H intrahepatic cholangiocarcinoma

Author	Age	Sex	TMB (muts/Mb)	PD-L1 expression	Testing method	MMR Gene variant	MMR deletion by IHC	Germline testing for MMR	CA19-9 (U/mL) while starting ICI	Therapeutic effect of ICI	Duration of survival after starting ICI (month)
Our case	65	M	29	Low	F1	MLH1 variant	MLH1, PMS2	Normal	85,964	PR	30 (alive)
Kai	71	M	Not tested	Not tested	MSI kit	Not tested	Not tested	Not tested	37	CR	22 (alive)
Toshida	70	M	Not tested	Not tested	MSI kit G360	Normal	Not tested	Not tested	48,321	PR	10 (alive)
Naganuma	60	M	Not tested	Not tested	Not mentioned	Not tested	Not tested	Not tested	Not mentioned	PR	13 (alive)
Nakamura	69	M	Not tested	Low	MSI kit	Not tested	Not tested	Not tested	1053	PR	7 (alive)
Ikeda	50	M	Not tested	Not tested	MSI kit	Not tested	Not tested	Not tested	2.5	PR	13 (alive)
Matsubayashi	43	M	10.09	Not tested	MGPT MSI test	Not tested	Proficient MMR	Not tested	41	PR	Not mentioned

*MSI-H* microsatellite instability-high, *MMR* mismatch repair, *TMB* tumor mutation burden, *IHC* immunohistochemistry, *ICI* immune checkpoint inhibitors *F1* FoundationOne CDx, *M* male, *CR* complete response, *PR* partial response

To date, MSI-H, TMB-H, and PD-L1 have been studied as predictors of the therapeutic efficacy of immune checkpoint inhibitors. Based on the KEYNOTE158 trial results, MSI-H has been demonstrated to be a marker of reasonable clinical promise [4,16]; however, PD-L1 expression has not necessarily been reported to be associated with efficacy. PD-L1 is not a predictor of the therapeutic efficacy of durvalumab, which was recently approved for the treatment of biliary tract cancer [3]. In our and Nakamura’s cases, a partial response was obtained with pembrolizumab treatment, despite low PD-L1. The importance of PD-L1 immunostaining may differ depending on the organs involved.

The KEYNOTE177 trial reported that pembrolizumab was less effective in patients with MSI-H colorectal cancer who had *KRAS* mutations, a strong driver gene, than in those without mutations [18]. Among the case reports, this and Toshida’s cases are the only ones in which comprehensive cancer genomic profiling was performed along with MSI testing [12]. Both patients showed partial responses to pembrolizumab, despite being *KRAS* mutation-positive. Although the number of cases was small, it was assumed that MSI-H intrahepatic cholangiocarcinoma may have a good therapeutic effect, even with KRAS mutations.

In this case, MLH1 protein deficiency was identified as the cause of MSI-H, which was consistent with the CGP genomic data showing MLH1 pathogenic mutations. Germline testing is recommended for MLH1 variants as presumed germline pathogenic variants^19–24^. Germline testing revealed no aberrant variants, indicating the absence of Lynch syndrome. If Lynch syndrome is suspected due to family or medical history, or if a gene mutation for which germline testing is recommended is detected in CGP testing, germline testing should be actively considered.

## Conclusion

We encountered a case of MSI-H intrahepatic cholangiocarcinoma caused by somatic MLH1 mutations, in which pembrolizumab was remarkably effective.

## Data Availability

Not applicable.
